# Effect of glare illusion‐induced perceptual brightness on temporal perception

**DOI:** 10.1111/psyp.13851

**Published:** 2021-05-25

**Authors:** Yuya Kinzuka, Fumiaki Sato, Tetsuto Minami, Shigeki Nakauchi

**Affiliations:** ^1^ Department of Computer Science and Engineering Toyohashi University of Technology Toyohashi Japan; ^2^ Electronics‐Inspired Interdisciplinary Research Institute Toyohashi University of Technology Toyohashi Japan

**Keywords:** glare illusion, perceptual magnitude, pupil diameter, pupillary light reflex, pupillometry, temporal perception

## Abstract

Temporal perception and the ability to precisely ascertain time duration are central to essentially all behaviors. Since stimulus magnitude is assumed to be positively related to the perceived duration from the early days of experimental psychology, most studies so far have assessed this effect by presenting stimuli with relatively different intensities in physical quantity. However, it remains unclear how perceptual magnitude itself directly affects temporal perception. In this study (*n* = 21, *n* = 20), we conducted a two‐interval duration‐discrimination task adapting a glare illusion (a visual illusion that enhances perceived brightness without changing physical luminance) to investigate whether the temporal perception is also influenced by perceptual magnitude. Based on the mean difference in the point of subjective equality derived from a psychometric function and pupil diameter, we found that temporal perception is influenced by the illusory brightness of glare stimuli. Interestingly, the perceived duration of the apparently brighter stimuli (glare stimuli; larger pupillary light reflex) was shorter than that of control stimuli (halo stimuli; smaller pupillary light reflex) despite the stimuli remaining physically equiluminant, in contrast with the well‐known "magnitude effect." Furthermore, this temporal modulation did not occur when the physical luminance of the stimuli was manipulated to match the illusory‐induced magnitude. These results indicate that temporal processing depends on the confluence of both external and perceived subjective magnitude and even illusory brightness is sufficient to affect the sense of duration; which may be explained by the internal magnitude decrease of the glare stimuli due to pupillary constriction decreasing the light entering the eye.

## Abstract 2

Our findings suggest a new viewpoint on the positive relationship between temporal perception and stimulus magnitude, in demonstrating that temporal processing depends on the confluence of both external and perceived internal magnitude. We provide evidence that illusory brightness induced by the glare‐illusion also influences the perceived duration which may be explained by the size of the pupil.

## 1 INTRODUCTION

Although time can be measured in objective standardized units, humans have no specific receptor to directly perceive time flows, neither do they have any mechanism to produce an accurate pulse such as the quartz resonators observed in digital clocks (Muller & Nobre, 2014).

Since the perception of time is fundamental to our daily lives and the ability to precisely time the duration of temporal intervals central to essentially all behaviors, temporal perception has been a focus of research since the early days of experimental psychology (Grondin, 2010). Although several models of temporal perception have been proposed, the underlying neuronal mechanisms remain unclear (Matthews & Meck, 2016). To answer this question, psychophysical and behavioral techniques have been employed to reveal how non‐temporal cognitive information modulates the subjective perception of time. Temporal illusions occur when the subjective perceived time duration does not faithfully represent its physical duration of an interval, which is assumed to be positively related to the magnitude of the stimuli (Eagleman, 2008; Rammsayer & Verner, 2014; Walsh, 2003).

Numerous studies have reported temporal illusions due to the physical magnitude of the stimulus by conducting temporal perception tasks with stimuli associated with different sensory inputs. For instance, a longer judged duration (temporal‐overestimation) as a function of stimulus magnitude has been demonstrated by non‐temporal stimulus attributes such as stimulus size (Rammsayer & Verner, 2014; Xuan et al., 2007), auditory loudness (Matthews et al., 2011), stimuli velocity (Makin et al., 2012), visual contrast (Benton & Redfern, 2016), numerosity (Vicario, 2011; Xuan et al., 2007), stimulus regularity (Sasaki & Yamada, 2017), or flicker frequency (Herbst et al., 2013) in the visual and auditory domains. Furthermore, these magnitude effects have been generalized to other modalities, such as the tactile, by electrical and vibrotactile stimulation (Williams et al., 2019).

Although many previous findings have generally indicated that the subjective duration of a given interval correlates positively with stimulus magnitude ("more‐is‐longer" account), it is equally important that this subjective duration is modulated as well by the relative and not absolute magnitude of the stimulus (Gomez & Robertson, 1979; Matthews & Meck, 2016). Gomez & Robertson showed that a large visual stimulus is judged as longer when compared to small objects, but only when object size varied within the session and participants could explicitly compare their sizes (Gomez & Robertson, 1979). This may be due to the fact that perceptual representations of the magnitude depend on the confluence of external stimulation and internal processing. For internal processing, the allocation of processing capacity, memory, and even mental effort are considered as factors modulating temporal perception through the final perceptual magnitude (Block & Gruber, 2014; Matthews & Meck, 2016). These interactions determine the final perceptual clarity, which can explain how increased stimuli intensity may lead to an overestimation of subjective temporal perception.

Ono and Kawahara reported altered perceived duration of an Ebbinghaus illusionary‐size‐varied object, also indicating the importance of the magnitude effect. Their results showed that the perceived duration for apparently larger stimuli was longer than the apparently smaller standard duration stimuli; furthermore, a bidirectionality effect was reported, where longer perceived stimuli were relatively perceived as larger (Ono & Kawahara, 2007). Non‐temporal factors such as physical and perceptual stimulus magnitude are known to affect subjective temporal perception. However, most studies focusing on magnitude‐related temporal illusions induced by stimulus magnitude so far assessed the effect of temporal illusions by presenting stimuli with relatively different physical intensities. Therefore, it remains unclear how perceptual magnitude itself directly affects temporal perception, and more direct evidence is required to understand how perceptual magnitude effects can be explained by the prominent temporal perception models/frameworks.

As luminance is one of the conspicuous magnitude dimensions in visual sensation, luminosity is one of the most focused non‐temporal stimulus attributes part of a general principle that subjective duration is positively related to stimuli magnitude. The early scientific inquiry of the effect of stimulus luminance on perceived duration was conducted by Goldstone et al. In a series of studies, Goldstone et al. used a red light‐emitting diode (LED) in a duration comparison task and asked the participants to judge which of the two durations (comparison or standard) was longer. The comparison stimulus was judged to be longer when the luminance was more intense than the standard, indicating that higher illumination increased subjective temporal perception (Goldstone & Goldfarb, 1964; Goldstone et al., 1978). Several subsequent studies have replicated the luminance effect in temporal judgment tasks using a tachistoscope (Brigner, 1986; Long & Beaton, 1980).

More recently, Xuan et al. investigated whether judgments of duration are modulated by magnitude information in various dimensions, such as space, quantity and time (Xuan et al., 2007). In a Stroop‐like interference paradigm, participants judged whether the duration of the two continuous stimuli was longer. The results indicated that temporal accuracy was higher when luminance intensity and the to‐be‐measured duration were congruent in different temporal tasks, in other words, when the short‐presented stimulus was dim, and the longer stimulus bright. Importantly, this congruency effect was found in different magnitudes (e.g., number of dots, size of a square), indicating the existence of generalized and abstract components in magnitude representations (Xuan et al., 2007). Similar to other non‐temporal aspects of temporal illusions, the prominent relationship between luminosity and subjective temporal perception is also reported to be affected by the relative, perceptual representations of luminance (Casini & Macar, 1997). Notably, absolute and relative stimulus brightness both predominantly affect temporal perception. Matthews and Stewart conducted several experiments fluctuating both target and background stimuli luminance. Surprisingly, the effect of stimulus magnitude on temporal perception depends upon the background: against a high‐intensity background, dim stimuli were judged as longer, so were bright stimuli on dark backgrounds (Matthews et al., 2011). These recent studies raise the possibility that the perceptual representation of luminance magnitude, which modulates temporal perception, may also depend on the interplay between external stimuli intensity and cognitive internal processing. Therefore, clarification of whether and how the perceptual intensity of brightness contributes to temporal perception should provide a key constraint on any model of the human perception of time.

**FIGURE 1:**
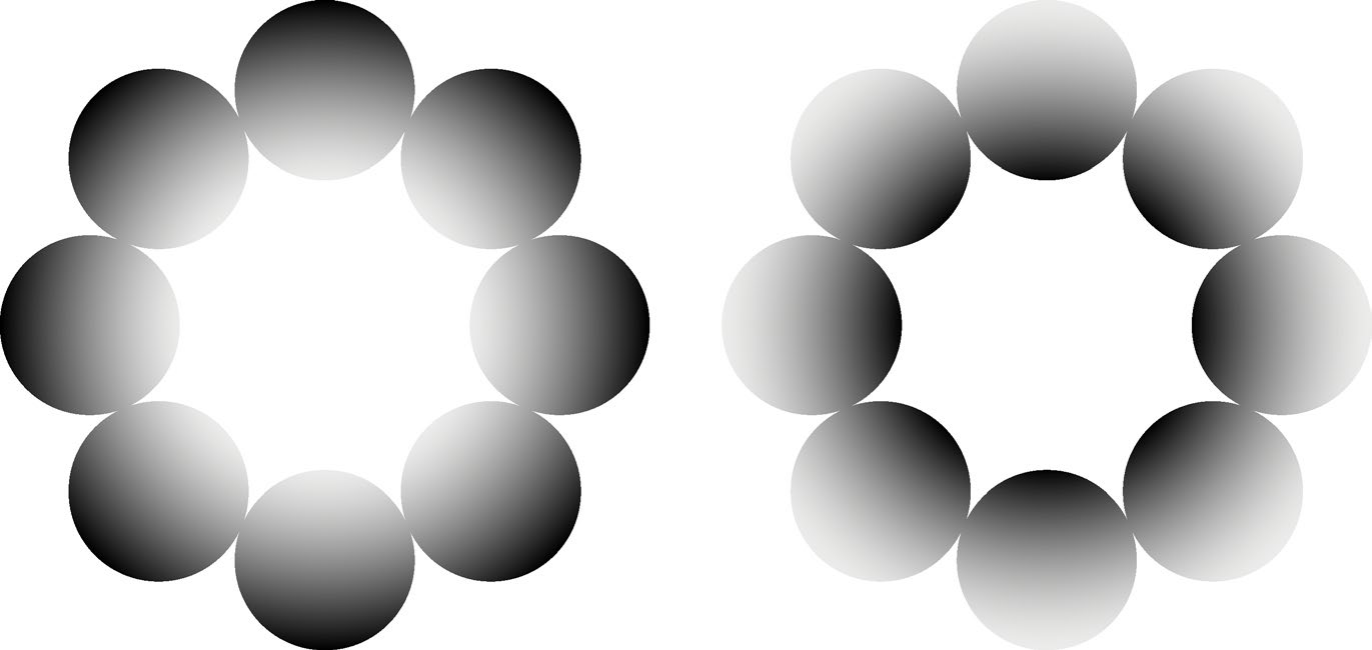
Glare illusion and halo stimuli. Both the glare illusion (left) and halo stimuli (right) consist of luminance gradient circles that either converge toward the pattern's center or toward the periphery (inducers). The central region of the glare illusion typically appears brighter than the background and the halo stimulus despite them being equiluminant. Note that the luminance in this figure differs from the stimuli used in our study

To examine this topic, we combined the perceptual luminance magnitude effect on temporal perception and glare illusion stimuli during pupillometry recording. The glare illusion is an optical illusion in which we perceive the central region to be brighter regardless of the actual luminance in the center region (Figure 1) (Agostini & Galmonte, 2002; Zavagno, 1999). The glare illusion is known as a robust illusion, with recent psychophysical studies showing that the illusion enhances the perceived magnitude of the brightness by 20% to 35% compared to the control stimuli (Tamura et al., 2016; Yoshida et al., 2008).

Additionally, pupil diameter is known to reflect not just the physical luminance of the environment but various physiological internal states as well as the subjective brightness derived from the stimuli (Binda et al., 2013; Laeng et al., 2012; Mathot, 2018; Naber & Nakayama, 2013). Accordingly, several studies have reported pupil diameter to also reflect the perceptual brightness conveyed by the glare illusion, despite the physical luminance being identical to the control stimuli (Laeng & Endestad, 2012; Laeng et al., 2018; Suzuki et al., 2019; Zavagno et al., 2017).

Therefore, in this study, we measured the perceived interval duration of visual objects whose perceived brightness was altered by the glare illusion while undergoing pupil diameter recording as an index of perceptual brightness. Additionally, the eye‐movement recordings, which were simultaneously conducted with pupillary recording, enabled us to examine the effect of saccadic eye movements on temporal illusions since Morrone et al. reported that a saccade during visual stimuli presentation leads to temporal compression (Morrone et al., 2005). This experimental design aimed to examine whether temporal illusions occur by glare illusions, where physical intensity is identical while perceptual brightness differs (Experiment 1). In addition, a control study was conducted to clarify whether the temporal modulation effect happens only with illusory luminance, and whether an actual physical luminance manipulation equivalent to the glare and halo also results in temporal illusions (Experiment 2). If the temporal perception process mainly relies on representation after the occurrence of the perceptual brightness illusion, the perceived interval duration of the glare illusion should be longer than that with halo stimuli due to the perceptual magnitude increment. If, however, the magnitude process is independent from the internal process, the perceived duration should remain the same since the physical luminance of both stimuli is identical. In other words, we hypothesized that the perceived duration of the glare illusion would be longer than with the halo stimuli due to the greater magnitude of internal representations.

## 2 EXPERIMENT 1

### 2.1 Method

#### 2.1.1 Participants

All experimental procedures and methods were in accordance with the ethical principles outlined in the Declaration of Helsinki and approved by the Ethics Committee for Human Research at the Toyohashi University of Technology. The experiment was strictly conducted in accordance with the approved guidelines of the committee. Informed written consent was obtained from participants after explaining the procedural details to them. Twenty‐one Japanese students (20 men, 1 woman; age range: 21–25 years (*M* = 21.7; *SD* = 1.20)) took part in the experiment.

None of the participant's eye movement data were excluded from pupil analyses since the trial rejection ratio did not exceed the criteria of 50% after interpolation in the pre‐processing phase. In the behavioral data analysis, trials where the reaction time (RT) > 10 s were excluded from analysis assuming low task performance. Only a limited proportion (0.13%) of trials were rejected by this criterion. All participants had a normal or corrected‐to‐normal vision and no participants reported color vision deficiency.

#### 2.1.2 Stimuli and apparatus

The task was conducted in a shielded, darkroom using MATLAB 2016a (The MathWorks, Natick, MA, USA) and a MATLAB toolbox, Psychtoolbox 3 (Brainard, 1997; Kleiner et al., 2007; Pelli, 1997). Instructions and stimuli were presented on an LCD monitor (Display++, Cambridge Research Systems Ltd, Rochester, UK) with a resolution of 1,920 × 1,080 pixels and a refresh rate of 120 Hz. As we gave importance to the equivalence of physical stimulus properties, colorimetric and spectro‐radiometric calibration was conducted in advance for linear light output (SR‐3AR, TOPCON, Tokyo, Japan). The eye‐tracker was placed below the presentation display, centered on the participant. The participant's head was placed on a chin rest at a viewing distance of 70 cm from the screen. All behavioral responses were performed using a numeric keypad with unnecessary keys removed.

In the temporal discrimination task, we used the glare illusion stimulus, which consists of an 8‐circular pattern arranged in a circular shape with luminance gradations converging to the central white area and the halo stimulus with the reverse luminance gradient (see Figure 1 for an example of the glare/halo stimuli; Note that the luminance in the figure differs from the stimuli used in our study). The achromatic gradation of the circular pattern in both stimuli changed progressively from 0.74 to 82.09 cd/m^2^ in luminance (i.e., the luminance on the inner and outer region of each circle was 82.09 and 0.74 cd/m^2^ respectively for the glare stimuli), a result of selected RGB values (*R* = 1.0, *G* = 1.0, *B* = 1.0), (*R* = 170.0, *G* = 170.0, *B* = 170.0). The background and central region of stimulus luminance also remained constant at 54.17 and 82.09 cd/m^2^, respectively, in the achromatic color (*x* = 0.3127, *y* = 0.329 in the CIE1931 color space). Both stimuli were presented with a visual angle of 12.10 degrees. During stimuli presentation, a small fixation cross was located in the center of the screen at a visual angle of 0.3 degrees for pupil diameter recording.

The pupillary response was recorded binocularly with an eye tracker (EyeLink 1000 Plus, SR Research, Oakland, Canada) at a sampling rate of 500 Hz. A five‐point calibration was performed prior to each session of the duration discrimination task.

#### 2.1.3 Procedure

**FIGURE 2:**
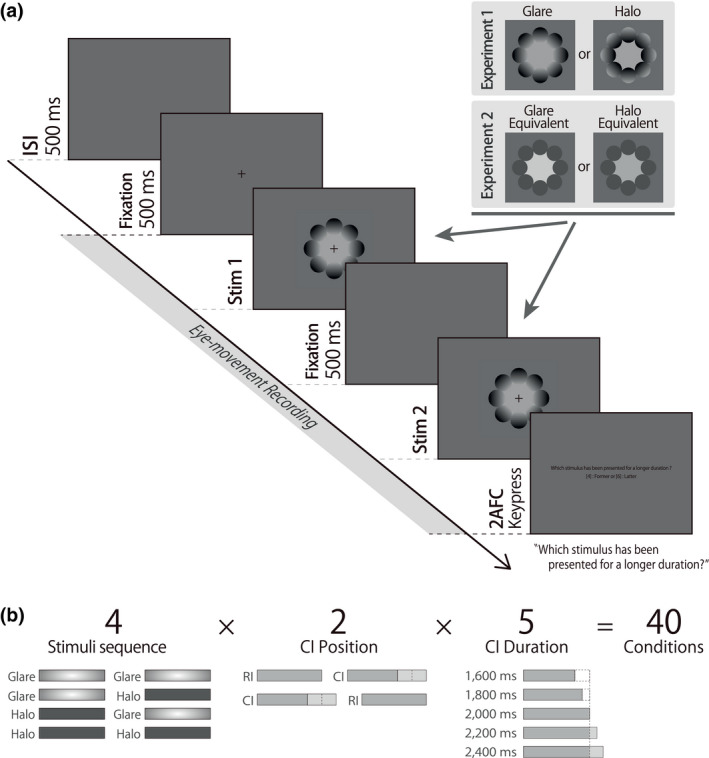
Example of the experimental protocol for one trial. (a) Sequence of one trial in the experiment. Stim1 and Stim2 refer to either the reference interval (RI) or comparison interval (CI). In Experiment 2, the two stimuli were replaced with subjectively equivalent uniform stimuli determined by an adjustment task. (b) Illustration of experimental conditions

The procedure of the duration discrimination task was based on a recent temporal perception study by Thönes et al. (2018), with 320 trials of a two‐interval duration discrimination task conducted over four sessions. Figure 2a shows the protocol for one trial in each session.

The eye‐tracker was calibrated prior to each session using a standard five‐point calibration, each session lasting approximately 10 min. In the two‐interval duration‐discrimination task, either the glare illusion stimuli or halo stimuli were presented continuously on the screen followed by the 500 ms inter‐stimulus interval (ISI). A fixation cross with a visual angle of 0.3 was continuously displayed at the center while eye‐movement recordings were performed. On each trial, one stimulus was presented for 2,000 ms (reference interval; hereinafter called RI), while the duration of the other stimuli varied between 1,600 to 2,400 ms in five steps (comparison interval; hereinafter called CI); separated by a blank interval of 500 ms. After the presentation of the two stimuli, the participant indicated which stimulus had been presented for a subjectively longer duration by a numeric keypad, with "4" indicating the former stimuli and "6" for the latter stimuli (two‐alternative forced‐choice without an option to indicate equal duration). No feedback was provided. The order of temporal positions was randomized and counterbalanced across sessions using a within‐subjects design. Figure 2b shows the combination of the 40 conditions in this study. Four different stimuli sequences were presented (glare‐glare, glare‐halo, halo‐glare, halo‐halo). The experimental condition was fully crossed by three experimental factors (stimuli sequence, CI position, and CI duration). Each condition was presented in 8 trials, resulting in a total of 320 trials per participant.

Prior to the main task, participants received a short practice session of randomly selected 10 trials, and sufficient breaks between the four sessions. Temporal judgment strategies such as counting‐up or other rhythmic activity which are reported to increase temporal sensitivity (Grondin et al., 2004) were not specifically instructed, although their use was not restricted.

### 2.2 Data analysis

#### 2.2.1 Eye movement measurement data

The timing of blinks during eye movement measurements was not specified to the participants; thereby, blinks were interpolated before analysis using cubic‐spline interpolation (Mathôt, 2013) in MATLAB 2018b. Trials that contained additional artifacts, computed by thresholding the peak changes on the velocity of the pupillary response, were excluded from the analysis. Pupil size was generated by the eye‐tracker device in arbitrary diameter units (EyeLink values). In the time‐course analysis, the pupil diameter at stimulus onset in each trial was normalized relative to the baseline pupil size, following the smoothing of each data point with ±10 sampling points. Baseline pupil size was derived as an average of data collected during the fixation period prior to each stimulus onset from −50 to 0 ms.

Saccades were detected by the velocity‐based algorithm proposed by Engbert and Kliegl (2003), computed by MATLAB 2020b after blink detection. The onset and termination of each saccade were determined by the timing when gaze velocity exceeded 30 degrees/s. The number of saccades per trial was normalized by the reference interval duration (2,000 ms). Bayes factor BF_10_ was computed by the Bayesian *t*‐test as an indicator to interpret the effect of the null hypothesis on the saccade rate.

#### 2.2.2 Behavioral data

The proportion of CI stimuli judged longer was calculated from the responses at each duration CI step. To estimate psychometric functions, the responses for each individual were modeled by fitting the logistic psychometric function using Palamedes toolbox for MATLAB (Prins & Kingdom, 2018). Threshold and slope were set to free parameters, although if any of the psychometric functions for each stimulus sequence condition could not be fit accordingly, all data for the applicable participants were excluded from the subsequent statistical analysis. After psychometric function fitting, the model was then used to compute the point of subjective equality (PSE) and just noticeable difference (JND) for each stimuli sequence condition (G‐G, G‐H, H‐G and H‐H; "G" and "H" for glare and halo, respectively, representing the CI‐RI combination). The PSE of the level of duration continuum at which the subjective temporal perception of the comparison stimuli is identical to the duration of the reference stimuli (RI), was computed to compare the effect of differences in perceptual brightness. R for Mac OS X version 3.5.1 and an analysis of variance function that runs on R language (anovakun version 4.8.2), was used for all statistical analyses. Pairwise comparisons of main effects were corrected for multiple comparisons using the Shaffer's MSRB (Modified Sequentially Rejective Bonferroni) and the significance level was set to *p* < .05 for the analysis of variance (ANOVA). In the ANOVA, partial *η* ($\eta_{p}^{2}$) is reported as a measure of association strength (effect size). In addition, Bayes factor BF_10_ was computed by the Bayesian repeated‐measures ANOVA as an indicator to interpret the effect of the null hypothesis.

#### 2.2.3 Mediation analysis

JASP 0.14.1 (JASP‐Team, 2020) was used to examine the direct and indirect effects in mediation analysis, implementing the bootstrapping procedure. The statistical significance of the mediating variable was investigated using 3,000 bootstrap samples to generate 95% confidence intervals of the indirect effects and examined if the interval is not straddling zero. Stimuli condition was used as a dummy variable (0: glare; 1: halo) and all regression coefficients standardized.

### 2.3 Results

#### 2.3.1 Duration judgment

**FIGURE 3:**
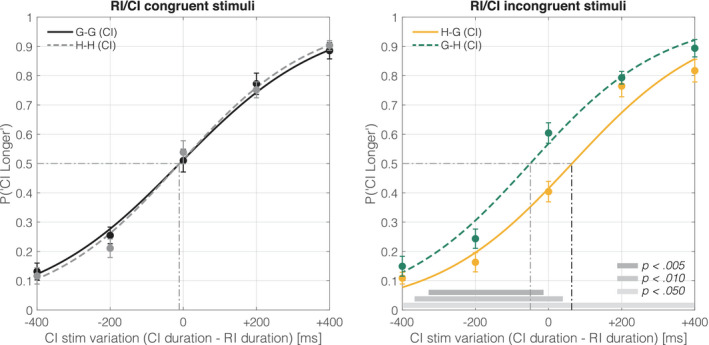
Mean psychometric functions. Left panel: psychometric function fitting by comparison interval (CI)‐longer proportion in congruent stimuli sequence. Right: Same as the left panel but for incongruent stimuli sequence. A rough indication of the significant *t*‐test domain is shown at the bottom. CI, comparison interval; G, Glare; H, Halo; RI, reference interval

In the two‐interval duration discrimination task, participants were instructed to indicate the longer stimuli of either the RI (in which the duration is fixed to 2,000 ms) or CI (1,600 to 2,400 ms in steps of 200 ms). Each probability was used toward psychometric function fitting by each duration step of the CI to compute the PSE and JND. Figure 3 represents the mean psychometric function computed by the average of all participants with a longer CI proportion in each CI duration difference condition (duration difference was calculated by subtracting RI duration from CI duration).

**FIGURE 4:**
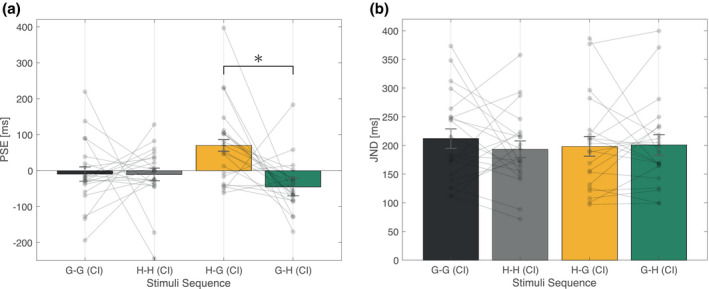
Mean psychophysical function properties (Experiment 1). (a) Mean PSE as a function of stimuli sequence. (b) Mean JND as a function of stimuli sequence. Error bars in both figures indicate standard error of the mean. *Statistically significant (*p* < .05) differences in the analysis of variance and post hoc testing. The semi‐transparent dot indicates the mean value for each participant data. CI, comparison interval; G, Glare; H, Halo; JND, just noticeable difference; PSE, point of subjective equality

In Figure 3, each solid line represents the fitted psychometric function. Each label indicates RI and CI stimuli, respectively (e.g., H‐G represents the RI stimuli being halo and the CI stimuli being glare). Dashed vertical lines represent the PSEs in each condition. The achromatic horizontal line at the bottom represents the domain with a significant difference as observed in the fitted function by *t*‐test analysis, for reference purposes. Note that no statistical correction to account for multiple comparisons was performed in this *t*‐test analysis. From the estimated psychometric function, a relative shift of the curve was observed in the incongruent stimuli comparison, whereas no shift was observed in the congruent stimuli comparison. The PSE and JND determined by the psychometric function are shown in Figure 4a,b, respectively.

Since CI position was not fixed and presented first or second with equal probability, a one‐way repeated‐measures ANOVA on the effect of stimuli sequence was performed. For the PSE, the effect of stimuli sequence was significant (*F*(3, 60) = 5.1919, *p* = .0030, $\eta_{p}^{2}$ = 0.2061), and therefore a post hoc *t*‐test was also conducted. The post hoc analysis showed that the two incongruent stimuli sequences, H‐G and G‐H, significantly differed as the PSEs were smaller when the CI stimulus was halo rather than glare, *t*(20) = 3.2719, adjusted *p* = .0229, whereas no other combination of stimuli sequences had significant differences. The shift of PSEs suggests an overestimation by halo stimuli compared with glare. Importantly, no overestimation was found in other stimuli sequences. In contrast to PSE, the main effect of stimuli sequence on JND, which is a measure of sensitivity, did not reach statistical significance (*p* < .05) and the effect size was relatively small (*F*(3, 60) = 0.3087, *p* = .8190, $\eta_{p}^{2}$ = 0.0152). Thus, we analyzed the present results with a Bayesian repeated‐measures ANOVA to statistically conclude no difference in the JND. The analysis indicated Bayes factor BF_10_ = 0.091, which is smaller than 1/10, providing strong evidence to support the null hypothesis (Dienes, 2014; Ortega & Navarrete, 2017). Importantly, the statistical analyses suggest no modulation of JND; in other words, temporal sensitivity did not differ by stimuli sequence.

#### 2.3.2 Pupillary response

**FIGURE 5:**
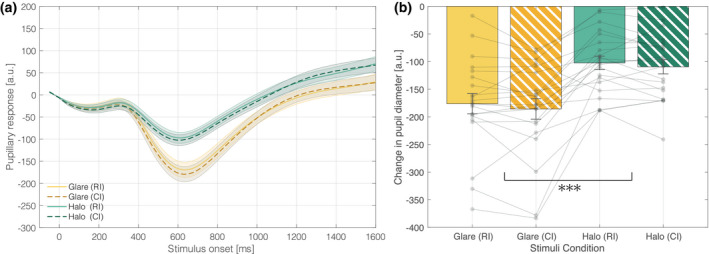
Pupillary response to each stimulus. (a) Mean change in pupil diameter from stimulus presentation. Solid and dashed lines represent pupillary responses by RI and CI stimuli, respectively. Error bars are standard error of the mean. Note that the *x*‐axis plot is limited to 1,600 ms, the shortest CI duration. (b) Mean PLR amplitude of each stimuli condition. PLR amplitude is computed as the minimum pupil diameter between 300 and 1,300 ms in the time domain. *** indicates statistically significant (*p* < .001) differences in the main effect of the ANOVA. The semi‐transparent dot indicates the mean value for each participant data. ANOVA, analysis of variance; CI, comparison interval; PLR, pupillary light reflex; RI, reference interval

We tracked changes in pupil diameter as an index of the perceptual brightness conveyed by the stimuli. All trials of glare and halo stimuli in both RI and all CI duration were averaged across conditions. Figure 5a shows the grand average of the pupil responses during a −50 to 1,600 ms stimulus onset under both stimuli (glare or halo) and duration (RI or CI) conditions.

The profile of a typical orienting response (Wang & Munoz, 2015) and the pupillary light reflex (PLR) were observed from the pupillary response. The peak pupil diameter of the PLR is depicted in Figure 5b, computed by the average of minimum pupil diameter between 300–1,300 ms in the time domain, given that PLR latency depends on the stimulus intensity, and has a peak generally around 500 ms (Ellis, 1981). As shown in Figure 5, the two‐way repeated‐measures ANOVA (stimuli condition × duration condition) evaluating the effect of peak pupil diameter revealed a significant main effect on the stimuli condition (*F*(1, 20) = 39.1142, *p* < .001, $\eta_{p}^{2}$ = 0.6617). However, duration condition did not reach statistical significance (*F*(1, 20) = 2.3175, *p* = .1436, $\eta_{p}^{2}$ = 0.1038). The results indicated a larger PLR in association with the glare stimuli compared with halo stimuli. As shown in Figure 5a, the mean minimum pupil diameter of the PLR induced by the glare illusion was 1.71‐times smaller than that by the halo stimuli. Assuming the pupil is a true circle, the pupil area at the PLR peak was only approximately 34% in association with glare stimuli compared to halo stimuli, despite the two stimuli being physically equiluminant. Lastly, as with pupillary response analysis, the number of saccades was computed in all glare and halo stimuli trials and then averaged across conditions. The average number of saccades per trial for glare and halo stimuli were 0.89 and 0.91, respectively. A paired samples *t*‐test between the number of saccades detected within the two stimuli conditions did not reach statistical significance (*p* < .05) and the effect size was relatively small (*t*(20) = −0.917, *p* = .370, *Cohen's d* = −0.200). Thus, we analyzed the present results with a Bayesian paired samples *t*‐test, finding no difference in the saccade rates. The analysis indicated a Bayes factor BF_10_ = 0.331, providing moderate evidence to support the null hypothesis (Dienes, 2014; Ortega & Navarrete, 2017).

#### 2.3.3 Mediation analysis

**FIGURE 6:**
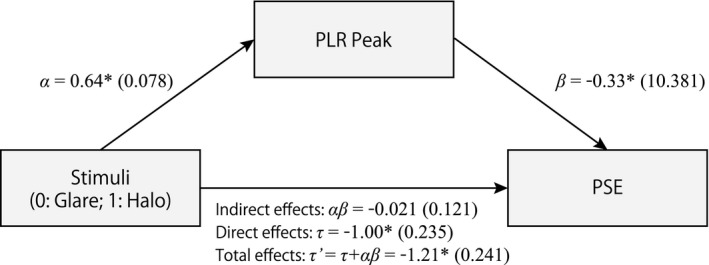
Schematic diagram of mediation analysis results. Depicted is the path diagram including regression coefficients of total and mediated effects of stimuli (glare/halo) on the PSE. Path values are standardized regression coefficients for the relationship with standard errors in parentheses. * indicates statistically significant (*p* < .05). PLR, pupillary light reflex; PSE, point of subjective equality

Results of the mediation analyses are presented in Figure 6. The standardized regression coefficient between stimuli (glare/halo) and PLR peak, computed by the minimum pupil diameter between 300–1,300 ms time domain, was significant, as was the standardized regression coefficient between PLR peak and PSE.

The significance of this indirect effect was evaluated using bootstrapping procedures. For each, 3,000 bootstrapped samples were used, and the 95% confidence intervals of the indirect effects were computed by determining the indirect effects at the 2.5th and 97.5th bias‐corrected percentiles. Path a and path b showed a significant regression; however, the bootstrapped indirect effect did not reach statistical significance (*αβ* = −0.021, *p* = .083), the 95% confidence interval ranged from −1.509 to −0.566, which did not include zero. A significant negative direct effect of stimuli on PSE was observed (Total effects; *τ'* = −1.21, *p* < .05). In addition, when the PLR peak was included as the mediator in the analysis, this coefficient was reduced but still statistically significant (Direct effects, *τ* = −1.00, *p* < .05).

### 2.4 Discussion

In this experiment, we investigated whether the temporal illusion of a visual stimulus is affected by the magnitude of perceptual brightness. Based on the prominent effects of magnitude on temporal illusions and a possibility that perceptual magnitude representations depend on the confluence of external stimulation and internal processing, one of our hypotheses was that the perceived interval duration of the glare illusion may be longer than that for halo stimuli due to the perceptual magnitude increment.

By means of a two‐interval duration‐discrimination task of glare and halo stimuli in the sub‐second range, psychometric function fitting was performed. Based on the analysis of psychometric functions, the mean difference in the PSE suggests that glare and halo stimuli were perceived to be of equal duration when glare stimuli were in fact physically 115 ms (5.7%) longer than halo stimuli, contrary to the initial hypothesis (see Figure 4a). In contrast, no difference was found in the JND (see Figure 4b). Additionally, no significant difference was found in the saccade rate between glare and halo stimuli, assessed to rule out the possibility that different stimulus‐induced saccade rates caused the temporal illusion (Morrone et al., 2005). The mediation analysis was conducted to further discuss the relationship between the temporal illusion effect and pupillometry response. Individual regression was significant; however, we did not find significant evidence for an indirect effect. In addition, we could not fully segregate the effect of illusory increased magnitude and equivalent magnitude increase by physical luminance manipulation on temporal illusions.

Therefore, to further focus on the effect of illusory luminance, an additional control study was conducted to a.) confirm that the apparent magnitudes of glare and halo do actually differ by the stimulus used in experiment 1, and b.) to clarify whether the temporal compression effect happens only with the illusory luminance and not physical luminance difference.

## 3 EXPERIMENT 2

### 3.1 Method

#### 3.1.1 Participants

In experiment 2, 20 Japanese students (20 men; age range: 22–26 years (*M* = 22.8; *SD* = 1.00)) were enrolled; six of these students had also participated in Experiment 1. In the behavioral data analysis, trials where the reaction time (RT) > 10 s were excluded from the analysis assuming low task performance (0.02% of the trials were rejected). All participants had normal or corrected‐to‐normal vision and no participants reported color vision deficiency.

#### 3.1.2 Stimuli and apparatus

The task was conducted in the same shielded, darkroom using MATLAB 2018b (The MathWorks, Natick, MA, USA) and a MATLAB toolbox, Psychtoolbox 3 (Brainard, 1997; Kleiner et al., 2007; Pelli, 1997) mentioned in Experiment 1. Glare and halo stimuli, sharing the same physical properties as those in Experiment 1, were used only in the adjustment task to compute the subjectively equiluminant uniform stimuli. The uniform stimulus consisted of an 8‐circular pattern (inducers) arranged in a circular shape identical to the glare/halo stimuli, except with a uniform luminance distribution of 39.88 cd/m^2^, a result of the mean RGB values used for the inducers of glare/halo stimuli (*R* = 85.5, *G* = 85.5, *B* = 85.5). The central region of the uniform stimulus luminance ranged from 44.31 to 114.28 cd/m^2^ based on the staircase method in the adjustment task in achromatic color (*x* = 0.3127, *y* = 0.329 in the CIE1931 color space). All glare, halo, and uniform stimuli were presented with a visual angle of 12.10 degrees throughout the experiment. Target (glare or halo) and uniform stimuli in the adjustment task were horizontally shifted at 7.70 degrees each from the center of the screen.

#### 3.1.3 Procedure

The procedure of the duration discrimination task was identical to Experiment 1. However, the to‐be‐measured stimuli were replaced with subjectively glare/halo‐equivalent uniform stimuli (see Figure 2a for one trial protocol).

To clarify whether the temporal illusion happens by illusory luminance, a behavioral luminance adjustment task was conducted in advance of the main duration discrimination task. In the adjustment task, a 2AFC (Two‐alternative forced‐choice) staircases method procedure was adapted to determine a subjectively‐equiluminant‐luminance‐value of glare and halo, respectively, for the center region of the uniform stimuli. Each trial in the adjustment task consisted of the presentation of the central fixation cross for 500 ms, followed by a simultaneous presentation of the target (glare or halo) and uniform stimulus for 3,000 ms. Subsequently, the participant reported whether the uniform stimuli were perceived brighter than the target (glare/halo) stimuli by using a numeric keypad. Based on the response and staircase procedure, the central region of the uniform stimulus luminance ranged ±75 RGB values from the baseline luminance of glare/halo (170 RGB value) in a step of 3 RGB values (approximately 1.40 cd/m^2^) per response. Trials of the staircase procedure were terminated when the number of reversals reached 8 times, or if the computed stimulus level continuously reached the limit for 3 times. Glare and halo were presented in 3 staircase trials, resulting in a total of 6 staircase trials per participant. The average of the final stimulus level was used toward the center region of the uniform stimuli in the duration discrimination task. The stimuli position was counterbalanced across participants, target stimuli order and initial stimuli level were randomized across trials using a within‐subjects design. Prior to each task, participants received a short practice session of randomly selected 10 trials and sufficient breaks between the tasks and the four sessions in the duration discrimination task.

### 3.2 Data analysis

All aspects of data analysis in duration judgment were analogous to Experiment 1. In addition to the temporal perception analysis, average adjusted luminance in the adjustment task was analyzed to compare the apparent magnitudes of the stimuli used in Experiment 1. The grand average of adjusted luminance was computed by the mean of each final stimulus level in the two conditions (glare and halo), and then averaged across participants. Finally, a paired sample *t*‐test was conducted on JASP 0.14.1 (JASP‐Team, 2020).

### 3.3 Results

**FIGURE 7:**
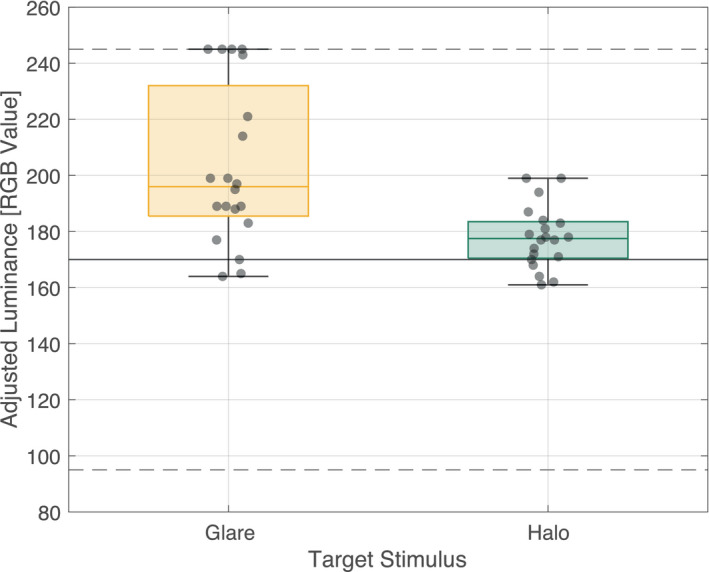
Mean adjusted luminance equivalent to glare/halo stimulus. Box plot of the mean determined RGB value for glare and halo stimulus. The central line in each box represents the median value and the box shows the interquartile range (the 25th percentile to the 75th percentile). The whiskers reflect the minimum and maximum. Upper and lower dashed and solid horizontal lines represent the limit of the staircase procedure and physically equiluminant value, respectively. The semi‐transparent dot indicates the mean value for each participant data

In the adjustment task, the luminance of the center area in the uniform stimuli was manipulated to match the subjective brightness of glare and halo stimuli. Figure 7 shows the distribution of the applied luminance value.

The mean luminance value for glare and halo was 203.1 (*SD*: 28.3) [RGB Value] and 177.9 (11.0) [RGB Value], respectively, and significantly different by a paired sample *t*‐test (*t*(19) = 4.617, *p* < .001, *Cohen's d* = 1.032) with a relatively large effect size.

**FIGURE 8:**
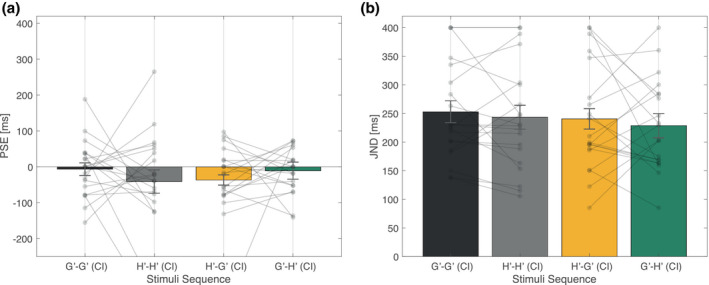
Mean psychophysical function properties (Experiment 2). (a) Mean PSE as a function of stimuli sequence. (b) Mean JND as a function of stimuli sequence. The semi‐transparent dot indicates the mean value for each participant data. CI, comparison interval; G', Glare‐equivalent; H', Halo‐equivalent; JND, just noticeable difference; PSE, point of subjective equality

The PSE and JND to the glare/halo‐equivalent stimuli sequence in the duration judgment task are shown in Figure 8a,b, respectively.

Again, a one‐way repeated‐measures ANOVA on the effect of stimuli sequence was performed. Contrary to Experiment 1, both the effect of stimuli sequence on PSE and JND did not reach statistical significance (PSE: *F*(3, 57) = 0.543, *p* = .655, $\eta_{p}^{2}$ = 0.028, JND: *F*(3, 57) = 0.611, *p* = .611, $\eta_{p}^{2}$ = 0.031). Therefore, we analyzed the present results with a Bayesian repeated‐measures ANOVA and found no significant differences in the PSE and JND. The analysis indicated BF_10_ = 0.134 and BF_10_ = 0.128 for PSE and JND, respectively, both smaller than 1/3, providing moderate evidence to support the null hypothesis (Dienes, 2014; Ortega & Navarrete, 2017). The statistical analyses suggest apparent duration and temporal sensitivity of the stimuli did not differ by stimuli sequence.

### 3.4 Discussion

In this second experiment, we conducted a duration discrimination task identical to Experiment 1, without eye‐tracking and stimuli replaced with the equivalent uniform stimuli to clarify whether the temporal compression effect happens with illusory luminance. The behavioral results from the adjustment task suggest that even though the luminance contrast of the glare stimuli used in this study was relatively low, the glare illusion was approximately perceived 14% brighter in physical luminance than halo stimuli (see Figure 7), as in previous reports (Agostini & Galmonte, 2002; Tamura et al., 2016). Furthermore, no difference was found in the PSE and JND (see Figure 8). However, this result contradicts previous studies considering that the perceptual representation of luminance can also influence perceived duration as explained in the "more‐is‐longer" account (e.g., Casini & Macar, 1997; Matthews et al., 2011). This effect can be simply due to the slight luminance difference of the glare and halo‐equivalent stimuli. In fact, the mean luminance difference of the center area was approximately 11.6 cd/m^2^, which is notably smaller than that obtained by previous studies conducted on a display or a tachistoscope (e.g., Matthews et al., 2011: 96.2 cd/m^2^, Brigner, 1986: 214.4 cd/m^2^). These results suggest that a physical luminance matched to the illusory‐induced magnitude is not sufficient to activate significant temporal modulation.

## 4 GENERAL DISCUSSION

In this study, we investigated whether the temporal illusion of a visual stimulus is affected by the magnitude of perceptual brightness rather than the physical magnitude of the luminance. In Experiment 1, we found that the glare illusion, which enhances apparent luminance, can evoke temporal modulation. In addition, Experiment 2 was conducted to clarify whether the temporal modulation effect happens only with illusory luminance. A PSE shift was not confirmed: an actual physical manipulation of luminance equivalent to the glare and halo did not activate significant temporal illusion. These results suggest stimuli with the same physical magnitude of luminance can evoke different subjective durations depending on how the stimuli‐related magnitude is perceived. In other words, although temporal illusions were observed by physically equiluminant stimuli, contrary to the widely reported "magnitude effect", the perceptual time of glare stimuli relative to halo stimuli was underestimated.

One explanation for this opposing glare stimuli‐associated underestimation in terms of the prominent magnitude effect is that the magnitude of the internal representation induced by the glare may decrease due to greater pupil constriction, thereby reducing the amount of incident light entering the pupil. Since our pupillary response is generally a function of retinal illuminance controlling the physical input from the ambient environment, the number and probability of photons captured by the retina decrease when the pupil is constricted even by glare illusions (Binda et al., 2013). In fact, the pupil area at the PLR peak induced by the glare stimuli was only approximately 34% in comparison with the halo stimuli, despite the physical equiluminance. Recently, Suzuki and colleagues reported that the amplitude of steady‐state visual evoked potentials (SSVEPs), electroencephalography (EEG) signals representing feature‐selective attention positively correlated with visual stimulus clarity, and surprisingly decreased in association with the glare illusion compared to control stimuli (Y. Suzuki et al., 2019). Furthermore, the study described the probable mechanism of this inhibited SSVEP as a causal relationship of glare illusion‐induced pupil constriction in the primary stages of visual processing and the decrease of light entering the pupil. Similarly, Bombeke et al. also suggested that pupil diameter differences directly modulate the magnitude of the feedforward response of V1 (Bombeke et al., 2016). Moreover, with respect to the retinal illuminance increase, a very recent study by Sulutvedt et al. reported even slight pupil dilations due to the act of Tropicamide (a medication used for pupillary dilation) treatment can result in an enhanced perceptual brightness (Sulutvedt et al., 2021). Since our perceptual representations of magnitude depend on the interaction between external stimulation and internal processing (Matthews & Meck, 2016), our results suggest the possibility that glare stimuli were perceived as shorter than halo stimuli, where the subjective magnitude decrease of glare stimuli was due to the constant pupillary constriction and was associated with a decrease in incident light entering the eye. Furthermore, our results and recent ﬁndings indicating the correlation between neural activities and pupil constriction may provide another explanation for temporal illusions through the well‐known temporal model, the coding efficiency model (Eagleman & Pariyadath, 2009). In this model, Eagleman and Pariyadath proposed that neural coding efficiency offers a basis of subjective time and pointed out various non‐temporal factors which expand perceptual duration also evoke larger neural responses (e.g., Matthews & Meck, 2016; Noguchi & Kakigi, 2006). Since glare stimuli are known to evoke lower EEG signals and responses of V1 due to the greater pupillary constriction, according to this model, halo stimuli may result in temporal overestimation by relatively larger neural activity. In other words, the temporal compression by the glare stimuli can be explained by the secondary effect of the pupillary constriction on the coding efficiency model, resulting in less neural activity in visual areas. Furthermore, the temporal illusion effect by the halo stimulus is assumed to be relatively weak, since its sensory magnitude is low compared to that of the glare stimulus, which has a reverse luminance gradient not inducive of brightness enhancement illusion and conspicuous pupillary constriction. Importantly, the perceived magnitude is also reported to positively relate to the apparent duration regardless of changes in physical magnitude. Considering coding efficiency model into account, a study by Murray et al. found that illusionary stimuli to be perceived as larger evoked greater cortical activity in V1 (Murray et al., 2006); this may explain the results by Ono and Kawahara that found that the same physical magnitude can extend subjective duration by illusionary larger objects (Ono & Kawahara, 2007), which also supports the supposition that subjective magnitude itself is sufficient to induce temporal illusions and explain the context‐dependency of non‐temporal effects on temporal perception. However, although Ono and Kawahara consider that temporal perception is also influenced by later processing related to visual illusions, considering bidirectional interactions in visual processing (Lollo et al., 2000), and the neural activity alteration resulting from pupillary constriction (Bombeke et al., 2016; Suzuki et al., 2019), the perceptual stage involved in temporal illusions is still speculative.

Some study limitations should be noted. First, there is a possibility that glare underestimation could be due to the stimuli' local contrast difference. Despite the fact each element used in the glare and halo stimuli is identical and the mean physical luminance is equiluminant, due to the angle of the luminance gradations halo, stimuli may have a larger visual contrast in the fovea. Benton and colleagues conducted a study to evaluate the effect of contrast on perceived duration, since neural activity in early visual areas is related to contrast (Benton & Redfern, 2016); they reported that an increase, in contrast, is related to temporal overestimation; however, the temporal illusion effect was relatively small. In our current study, a fixation cross was located in the center of the screen for pupillometry recording. Therefore, while the main stimuli were relatively small (12.10 degrees), participants could overestimate halo stimuli compared to the glare by the central stimulus contrast. Further, due to the ambiguous border by the stimulus gradient, we cannot fully deny the possibility that apparent size differed from the stimuli. Additionally, temporal illusions are often explained in terms of the attention state to the stimuli, since the increase of selective attention in temporal tasks is known to be associated with temporal sensitivity (Grondin et al., 2014). However, our analysis suggests no JND modulation in temporal judgments; thus, the effect of the contrast and attention process derived by the illusion is assumed to be relatively limited.

Second, the mechanisms of temporal processing are known to vary between sub‐second (milliseconds) and supra‐second (seconds to minutes) interval ranges (Lewis & Miall, 2003); since working memory is important for temporal processing such as duration comparisons of supra‐second intervals (Lee et al., 2009), the timing of sub‐second intervals are assumed to be a relatively automatic process and beyond cognitive control. Therefore, to further elucidate the perceptual magnitude effect by visual illusions in the early stages of visual processing, investigation in the sub‐second range should also be taken into consideration. Third, this study has focused on the temporal distortion and pupillary response by visual stimuli. However, the pupillary response is known to reflect many other cognitive factors since pupil diameter is determined by antagonistic activation in the autonomic nervous system (comprising the sympathetic and parasympathetic nervous system; Mathot, 2018) and used as an indirect measure of activity in the locus coeruleus (LC), a brainstem nucleus involved in noradrenergic transmission (Mathot, 2018). Considering this antagonistic effect, the increase in parasympathetic activity due to the pupillary constriction induced by the glare illusion may decrease sympathetic activity, which is associated with arousal, resulting in temporal compression by the glare stimuli. In addition, since some studies focused on the correlation between neuromodulation of the pupil size, the extent of LC activation, and subjective temporal perception in non‐human primates (Faber, 2017; Suzuki et al., 2016), further interdisciplinary considerations incorporating both psychology and neuroscience approaches will be needed to yield any fundamental findings to deconstruct the complex neurobiological process and the role of the LC and pupillary response on temporal perception.

In conclusion, based on the analysis of psychometric functions, mean difference in the PSE and pupil diameter, our results are the first to demonstrate that temporal perception is also influenced by illusory brightness in glare stimuli, indicating the possibility that temporal processing depends on the confluence of both external magnitude and perceived subjective magnitude. In our study, pupillary response was recorded as an index of perceptual magnitude, since pupil diameter (e.g., PLR) is known to reflect subjective brightness derived from the stimuli. The perceived duration of glare stimuli (apparently brighter; larger PLR) was shorter than that of halo stimuli (control stimuli), although the physical luminance remained equiluminant. This surprising temporal illusion by glare stimuli contrasts with the well‐known magnitude model assuming the positive correlation of the subjective duration of a given interval with stimulus magnitude. However, this inconsistency may be explained by the subjective magnitude decrease of glare stimuli due to the constant pupillary constriction decreasing the light energy entering the eye, consistent with the coding efficiency model by Eagleman and Pariyadath (Eagleman & Pariyadath, 2009). As glare stimuli evoked a greater pupil constriction resulting in an over 60% decrease in pupil area compared to halo stimuli, EEG signals and V1 responses may also decrease, thus replicating previous studies that lead to glare stimuli's temporal illusion. However, these links remain speculative at present and further studies regarding the relationship between pupillary responses including incident light amount and perceived duration are required.

## ETHICAL APPROVAL AND INFORMED CONSENT

All experimental procedures were in accordance with the ethical principles outlined in the Declaration of Helsinki and approved by the Committee for Human Research at the Toyohashi University of Technology. The experiment was strictly conducted in accordance with the approved guidelines of the committee. Informed written consent was obtained from participants after procedural details had been explained.

## OPEN PRACTICES STATEMENT

Neither of the experiments reported in this article was formally preregistered. Neither the data nor the materials have been made available on a permanent third‐party archive; all dataset generated during this study are included in this article and analyzed data are fully available from the corresponding author via email on reasonable request.

## CONFLICT OF INTEREST

The authors have declared that no competing financial interests exist.

## AUTHOR CONTRIBUTIONS

**Yuya Kinzuka:** Conceptualization; Data curation; Formal analysis; Investigation; Visualization; Writing‐original draft; Writing‐review & editing. **Fumiaki Sato:** Data curation; Writing‐review & editing. **Tetsuto Minami:** Conceptualization; Funding acquisition; Supervision; Writing‐review & editing. **Shigeki** **Nakauchi:** Funding acquisition; Supervision; Writing‐review & editing.
